# Smart colorectal endoscopic submucosal dissection: integrating traction, pocket creation and underwater dissection to enhance efficiency

**DOI:** 10.1055/a-2936-4129

**Published:** 2026-08-25

**Authors:** Pier-Anne Gaulin, Paul Carrier, Sophie Geyl, Jérémie Albouys, Romain Legros, Mathieu Pioche, Jérémie Jacques

**Affiliations:** 1 Hepatogastroenterology Department 36715 University Hospital Centre of LimogesLimogesFrance; 2 Gastroenterology DepartmentEdouard Herriot HospitalLyonFrance

**Keywords:** Endoscopy Lower GI Tract, Polyps / adenomas / ..., Endoscopic resection (polypectomy, ESD, EMRc, ...), Quality and logistical aspects, Training

Colorectal endoscopic submucosal dissection (ESD) remains technically demanding, particularly in the colon where limited scope maneuverability can make dissection challenging. Although ESD enables en bloc resection with optimal histological assessment compared with piecemeal endoscopic mucosal resection (EMR), its broader adoption—especially for large benign colorectal lesions—relies on improving procedural efficiency. 1​ In contemporary practice, these approaches are not mutually exclusive; rather, “smart” colorectal ESD relies on the pragmatic integration of complementary techniques to optimize traction, stability, and efficiency, even in expert hands. 2​ ​3​

**Fig. 1:**
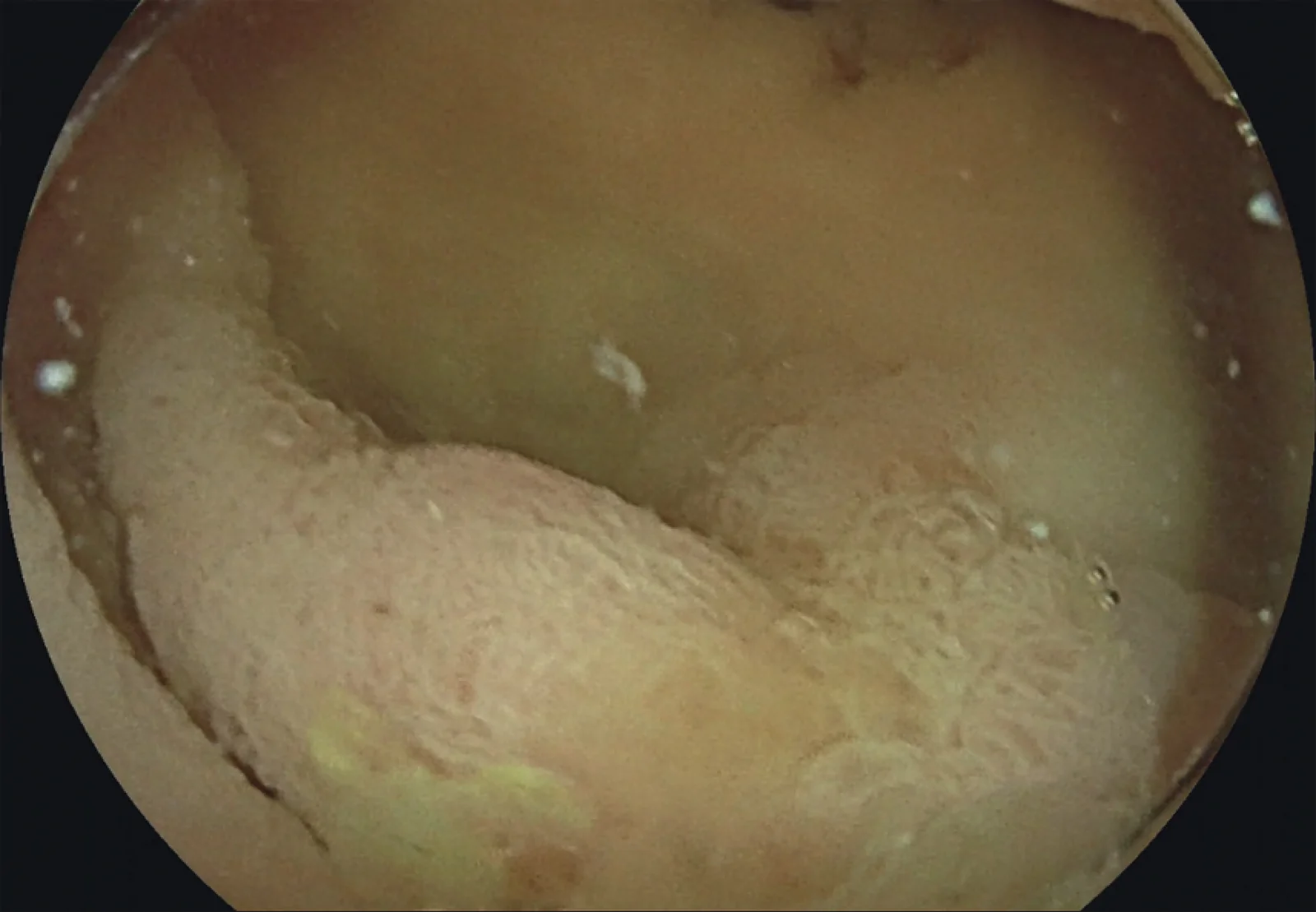
5 cm granular LST in the cecum.

We present this concept during ESD of a 5-cm granular laterally spreading tumor in the cecum, where scope maneuverability was particularly challenging ( Fig. 1 ).

**Fig. 2:**
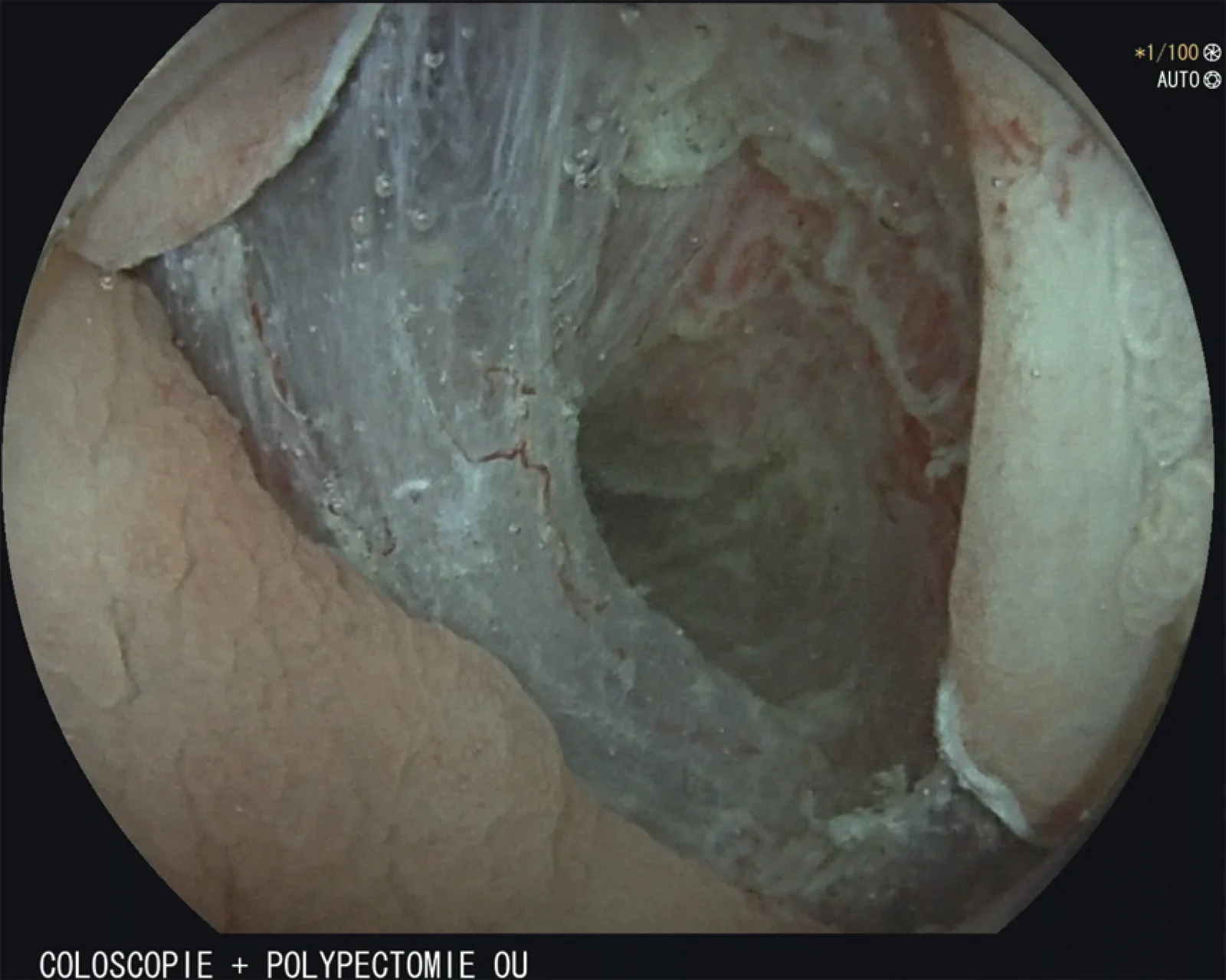
One additional loop of the traction is engaged to restore tension and facilitate dissection.

After submucosal injection with a viscous solution, circumferential incision and trimming were completed. Initial traction was applied to improve access to the submucosal plane using a multi-loop clip device, consisting of a clip preloaded with a five-loop elastic system enabling multipoint traction during dissection. A pocket-creation approach was then used to stabilize the endoscope and maintain a controlled dissection plane, given the limited maneuverability in this location. As the submucosal tunnel progressed, traction progressively decreased, making the completion of the lateral dissection more challenging. One additional loop of the traction system was engaged to restore tension on the lesion’s edges, improving exposure and facilitating resection ( Fig. 2 ).

En bloc resection was achieved in 45 minutes, yielding a 55×45 mm specimen with a 40-mm low-grade dysplasia and R0 margins ( Video 1 ).

**Video 1** Video template of colonic ESD.


This case highlights how the integration of complementary strategies—including underwater dissection to enhance visualization and electrosurgical efficiency, traction to improve submucosal exposure, and the pocket-creation method to stabilize the endoscope—combined with dynamic adjustment during dissection, can simplify complex colorectal ESD. Achieving efficient en bloc resection, even in challenging colonic locations, may support the broader adoption of ESD for large benign colorectal lesions. 4​

Endoscopy_UCTN_Code_TTT_1AQ_2AD
